# Association of triglyceride-glucose index and remnant cholesterol levels with liver fibrosis progression and disease severity in patients with non-alcoholic fatty liver disease: a cross-sectional study

**DOI:** 10.3389/fendo.2025.1686162

**Published:** 2025-11-24

**Authors:** Qiong Duan, Yue Wang, Juan Xu, Long Cheng, Wenqing Hong, Pinglu Feng, Wenrui Lu, Xihai Xu, Xu Zhang

**Affiliations:** 1 Department of Health Management Center, First Affiliated Hospital of Anhui Medical University, Hefei, China; 2 Department of Oncology, First Affiliated Hospital of Anhui Medical University, Hefei, China

**Keywords:** non-alcoholic fatty liver disease, triglyceride-glucose index, remnant cholesterol, severity, liver fibrosis

## Abstract

**Background:**

The associations of the triglyceride-glucose (TyG) index and remnant cholesterol (RC) with NAFLD severity (mild, moderate, and severe) and liver fibrosis remain unclear. This study examined these relationships and assessed the impact of TyG\RC on liver fibrosis.

**Methods:**

594356 participants undergoing annual physical and ultrasonic examinations were included. Standardized questionnaires collected clinical data, and venous blood samples were measured for complete blood count, liver function, and metabolic parameters. TyG index and RC values were calculated, and liver fibrosis assessed using the NAFLD fibrosis score (NFS) and aspartate aminotransferase-to-platelet ratio index (APRI). Participants were categorized into TyG and RC levels quartiles (Q1–Q4) to examine their association with NAFLD severity. Sensitivity analyses were conducted subsequent to multiple imputation application for missing values and outliers.

**Results:**

102599 NAFLD patients (75414 mild, 19412 moderate, 7733 severe) were included. RC exacerbated disease progression (odds ratio (OR) = 1.336, 95% confidence interval (CI): 1.163–1.537), whereas TyG index can inhibit it (OR = 0.436, 95% CI: 0.224–0.848). Compared with Q1 of TyG, participants in Q2 (OR = 0.897, 95% CI: 0.852–0.944) and Q3 (OR = 0.883, 95% CI: 0.839–0.930) inhibited the risk of NAFLD progression. Conversely, higher RC quartiles (Q2: OR = 1.114, 95% CI: 1.064–1.168; Q3: OR = 1.103, 95% CI: 1.048–1.149; Q4: OR = 1.251, 95% CI: 1.195–1.310) aggravated NAFLD progression. Additionally, sensitivity analyses yielded consistent results that were consistent with those of the initial analysis. Linear regression indicated that each 1-unit rise in TyG correlated with NFS (β = 0.407, 95% CI: 0.271–0.544) and APRI (β = 0.035, 95% CI: 0.034–0.037) increases, while a 1-unit RC increase corresponded to NFS (β = 0.791, 95% CI: 0.725–0.857) and APRI (β = 0.011, 95% CI: 0.010–0.012) elevations.

**Conclusion:**

The TyG index and RC may independently influence NAFLD progression and hepatic fibrosis. Elevated levels of both biomarkers contribute to fibrosis development, highlighting their utility in risk stratification and potential as therapeutic targets.

## Graphical Abstract

(TyG and RC are associated with NAFLD progression and liver fibrosis). 
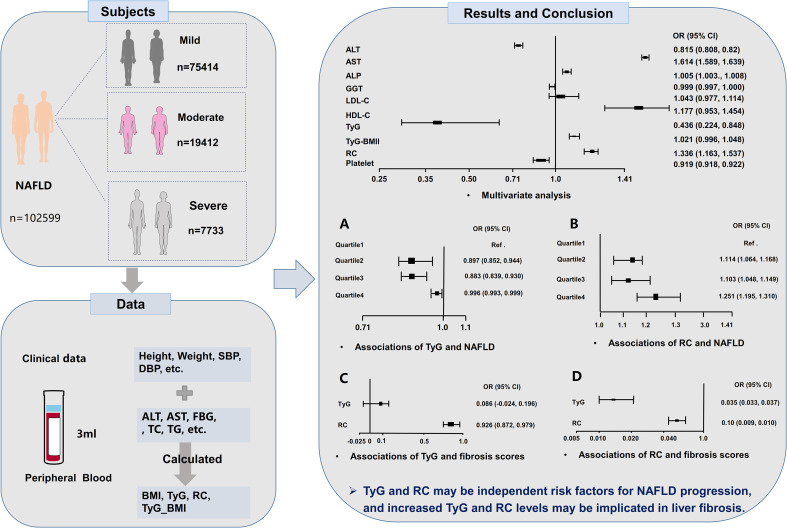

## 1 Introduction

Non-alcoholic fatty liver disease (NAFLD) represents a spectrum of chronic liver conditions ranging from hepatic steatosis to nonalcoholic steatohepatitis (NASH), fibrosis, cirrhosis, and ultimately hepatocellular carcinoma (1–3). With a global prevalence affecting approximately 25% of the adult population and a rising incidence among younger demographics, NAFLD presents significant public health challenges (2, 4). The current understanding of its pathogenesis is incomplete, and diagnostic as well as therapeutic options remain limited.

The pathogenesis of NAFLD involves complex metabolic disturbances as described by the ‘multiple-hit’ hypothesis, which includes insulin resistance (IR), chronic inflammation, oxidative stress, and excessive triglyceride deposition (5, 6). Elevated levels of plasma triglycerides (TG) and total cholesterol (TC) disrupt hepatic lipid metabolism, stimulating free fatty acids (FFA) overproduction (7). The subsequent uptake of FFA by hepatocytes exacerbates both IR and hepatic steatosis (7). Although the role of IR in the progression of NAFLD is well-established, its clinical and epidemiological applications require further investigation (8).

The triglyceride-glucose (TyG) index and remnant cholesterol (RC) are recognized as reliable indicators of lipid dysregulation and IR (9, 10). While some studies have associated TyG and RC with NAFLD, the evidence remains fragmented and inconclusive (10). Prior research is characterized by methodological limitations and restricted sample sizes (9, 11–14). Additionally, these studies also neglected to examine the relationship between TyG and RC across different NAFLD severity levels (mild, moderate, severe) or rigorously assessed their association with liver fibrosis (9, 11–14).

In light of the prognostic significance of NAFLD severity, this large-sample cross-sectional study investigates the associations of RC and TyG index across various disease stages of the disease and their potential role in liver fibrosis. Our findings contribute to the existing evidence by linking these accessible biomarkers to the progression of NAFLD.

## 2 Materials and methods

### 2.1 Design and population

This cross-sectional study was conducted at the First Affiliated Hospital of Anhui Medical University from April 2019 to December 2023. The study protocol received approval from the Clinical Research Ethics Committee of the First Affiliated Hospital of Anhui Medical University (Approval No. 20210026), and all participants provided written informed consent.

### 2.2 Diagnostic criteria, inclusion and exclusion criteria

The diagnosis of NAFLD required meeting all ultrasonographic criteria evaluated independently by two experienced sonographers (15). The diagnostic criteria included: (1) hepatic near-field echogenicity surpassing that of renal or plenic echogenicity, accompanied by far-field attenuation; (2) obscured intrahepatic duct architecture, rounded liver margins, and hepatomegaly; (3) absence of significant alcohol consumption, defined as >20 g/day for men or >10 g/day for women; and (4) exclusion of secondary causes of fatty liver etiologies, such as viral hepatitis and drug-induced liver injury.

The inclusion criteria were as follows: (1) Met the diagnostic criteria. (2) No prior history of fatty liver disease. (3) Completion of standardized liver ultrasonography. (4) Aged ≥ 18 years.

The exclusion criteria included: (1) Excessive alcohol consumption. (2) A history of NAFLD. (3) Viral hepatitis or malignant tumors. (4) Incomplete liver ultrasound evaluation.

### 2.3 NAFLD severity

The NAFLD severity grading (mild, moderate, severe) was based on standardized ultrasonographic criteria: (1) Mild: Slight diffuse increase in hepatic echogenicity with normal visualization of the diaphragm and intrahepatic vessel borders. (2) Moderate: Moderate diffuse increase in hepatic echogenicity with slightly impaired visualization of the diaphragm and intrahepatic vessels. (3) Severe: Marked increase in echogenicity with poor penetration of the posterior segment of the right lobe and poor or non-visualization of the hepatic vessels and diaphragm.

### 2.4 Data collection and measurements

Demographic and clinical parameters, including sex, age, height, weight, systolic blood pressure (SBP), and diastolic blood pressure (DBP), were systematically recorded. Measurements of Height and weight were conducted twice using a calibrated HNH-219 automated stadiometer/scale (Manufacturer, City, Country), with participants barefoot and wearing light clothing, and the average values were subsequently recorded. Blood pressure was measured twice by trained clinicians following 5-minute rest period, using a validated electronic sphygmomanometer (Model, Manufacturer) in accordance with the American Heart Association (AHA) guidelines. Fasting venous blood samples (3 mL) were collected and processed within two hours using a BC-5000 Auto Hematology Analyzer (Mindray, Shenzhen, China) for complete blood count analysis. The institutional central laboratory conducted liver function and metabolic panel assessments, which included alanine aminotransferase (ALT), aspartate aminotransferase (AST), total cholesterol (TC), triglycerides (TG), high-density lipoprotein cholesterol (HDL-C), low-density lipoprotein cholesterol (LDL-C), albumin, γ-glutamyl transpeptidase (γ-GGT), and fasting blood glucose (FBG), using standard automated biochemical analyzer (Hitachi).

### 2.5 Non-invasive biomarkers

Calculation of metabolic indices (3, 9):


BMI=weight (kg)/height ^2 (m)

$$\text{TyG}=\ln[\frac{\text{ TG }(\text{mg}/\text{dL})\;\times \text{ FPG }(\text{mg}/\text{dL})}{2}]$$

TyG_BMI=TyG ×BMI

RC=TC−HDL_C−LDL_C

### 2.6 Non-invasive liver fibrosis score

Non-invasive liver scoring systems are widely utilized in patients with NAFLD to identify individuals at risk for advanced fibrosis. This study employed the NAFLD fibrosis score (NFS) and the aspartate aminotransferase-to-platelet ratio index (APRI) to evaluate the risk of liver fibrosis (1, 16).


APRI=(AST/Upper limit of normal)  ×100 / platelets (10^9/L) 

NFS=−1.675+0.037×Age+0.094×BMI(kg/m^2)+1.13×Impaired glucose tolerance or diabetes(if yes is 1,No is 0)+0.99×AST/ALTratio−0.013×platelets(10^9/L)−0.66×albumin(g/dl)

### 2.7 Statistical analysis

Prior to conducting the analysis, an assessment for outliers was performed on continuous variables. Potential univariate outliers were identified both visually using boxplots and numerically using the interquartile range (IQR) method, where values below Q1-1.5 × IQR or above Q3 + 1.5 × IQR were flagged. All flagged outliers were double-checked for data entry errors and clinical plausibility. As the identified outliers were considered biologically plausible (e.g., extreme but possible laboratory values), they were retained in the primary analysis. To mitigate their excessive influence on regression models, multiple imputation was utilized to address skewed and missing data. Subsequently, multicollinearity diagnostics were conducted on the independent variables using the variance inflation factor (VIF), tolerance (Tol), condition index, and eigenvalues.

The normality of continuous data was assessed using the Kolmogorov-Smirnov test. Skewed data were reported as median (interquartile range) and analyzed using the Mann-Whitney U test. Categorical variables were compared using Pearson’s chi-square test. Ordinal regression was performed to examine factors contributing to different stages of NAFLD, while multivariate ordinal regression was applied to variables that were significant (*P* < 0.05) in the univariate ordinal analysis. In addition, sensitivity analyses were conducted by re-running the primary models after applying an imputation method for key continuous variables. Linear regression was used to assess the associations between TyG, RC, and liver fibrosis scores, with forest plots used to visualize significant factors. Three multivariate ordinal models were constructed: an unadjusted model (Model 1), a model adjusted for age and sex (Model 2), and a model additionally adjusted for BMI, SBP, and DBP (Model 3). All analyses were performed in R and SPSS, with *P* < 0.05 considered statistically significant.

## 3 Results

### 3.1 Demographic and clinical characteristics

**Figure 1:**
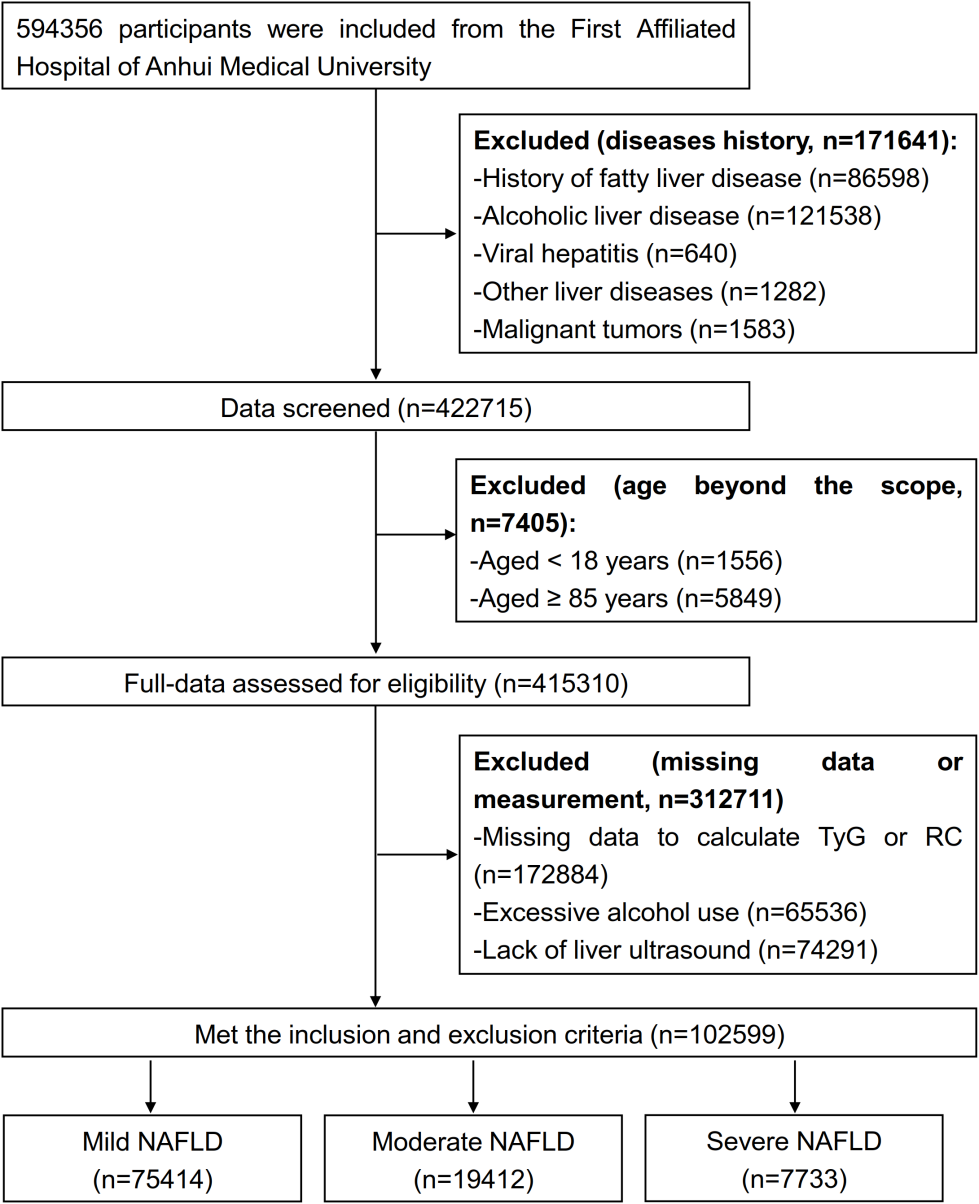
Flowchart of study cohort selection.

This study initially identified 594, 356 potential participants and removed 171, 641 diseased individuals, and 422, 715 individuals were excluded when we screened data; we assessed 415, 310 individuals’ data and excluded 312, 711 participants. Overall, 102, 599 patients with NAFLD met the inclusion criteria, including 75, 414 mild, 19, 412 moderate and 7, 733 severe patients, were enrolled after the screening process (**Figure 1**).

Mild patients with NAFLD patients exhibited a median age of 43.0 years (IQR 35.0, 53.0), compared to 60.0 years (54.0, 68.0) for moderate and 53.0 years (41.0, 62.0) for severe. The proportion of female patients was 25.7% in the mild group, 35.4% in the moderate group, and 29.3% in the severe group. The median RC values were 0.6 (0.3, 0.9) for both mild and moderate groups, and 0.5 (0.2, 0.9) for severe group, while all groups exhibited identical median TyG values of 1.6 (1.2, 2.0). Comprehensive clinical and demographic characteristics are shown in the **Supplementary Table 1**.

### 3.2 Determinant factors of patients with NAFLD

Prior to conducting the multivariate ordered regression analysis, all independent variables were assessed for multicollinearity. The results revealed that all VIF values were below 10 (**Supplementary Table 2**), all Tol values exceeded 0.1 (**Supplementary Table 2**), all condition indices were less than 30, and all eigenvalues were greater than 0 (**Supplementary Table 3**), suggesting the absence of multicollinearity among the independent variables.

**Figure 2:**
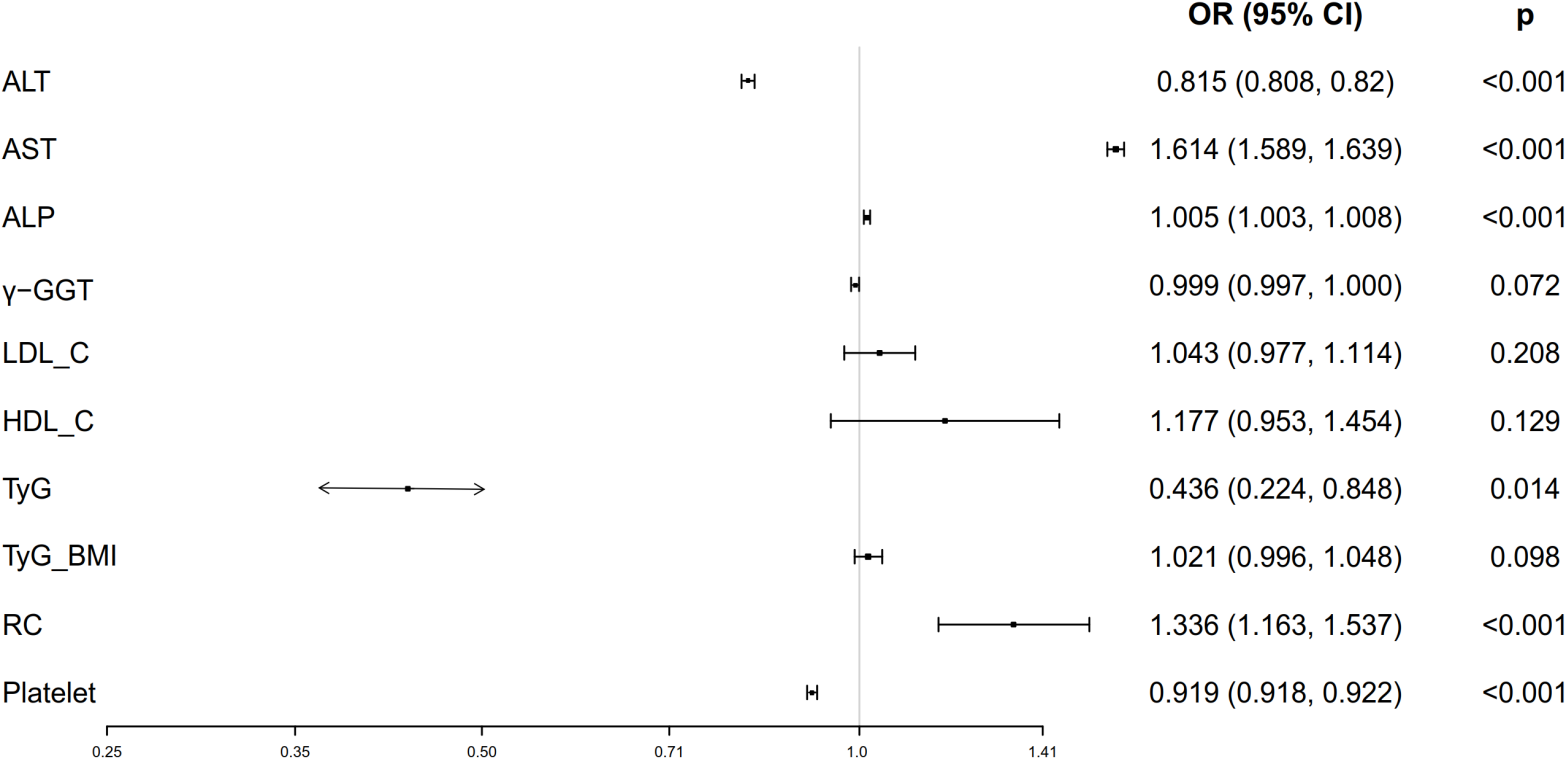
Forest plots of independent factors are associated with NAFLD (adjustment for age, gender, SBP, DBP and BMI).

Univariate ordinal regression analysis showed that ALT, AST, ALP, γ-GGT, LDL-C, HDL-C, platelet, TyG, TyG-BMI and RC were associated with the progression of NAFLD (**Table 1**). Subsequently, multivariate ordinal regression analysis revealed that AST (OR = 1.614 (1.589, 1.639), *P* < 0.001), ALP (OR = 1.005 (1.003, 1.0080, *P* < 0.001), LDL-C (OR = 1.043 (0.977, 1.114), *P* = 0.208), HDL-C (OR = 1.177 (0.953, 1.454), *P* = 0.129), and RC (OR = 1.336 (1.163, 1.537), *P* < 0.001) exacerbated the progression of NAFLD (**Table 1**, **Figure 2**), while ALT (OR = 0.815 (0.808, 0.82), *P* < 0.001), γ-GGT (OR = 0.999 (0.997, 1.000), *P* < 0.001), platelet (OR = 0.919 (0.918.0.922), *P* < 0.001), and TyG (OR = 0.436 (0.224, 0.848), *P* < 0.014) can inhibit its progression (**Table 1**; **Figure 2**).

**Table 1:** Determinant factors of NAFLD progression.

Variables	Univariate analysis		Model 1		Model 2		Model 3	
	OR (95% CI)	*P* for trend	OR (95% CI)	*P* for trend	OR (95% CI)	*P* for trend	OR (95% CI)	*P* for trend
Gender	1.423 (1.381, 1.465)	<0.001						
Age	1.09 (1.088, 1.091)	<0.001						
SBP	1.02 (1.019, 1.021)	<0.001						
DBP	1.007 (1.006, 1.008)	<0.001						
BMI	0.991 (0.987, 0.994)	<0.001						
Albumin	1.000 (0.999, 1.001)	0.496						
ALT	0.998 (0.997, 0.998)	<0.001	0.880 (0.876, 0.883)	<0.001	0.814 (0.808, 0.82)	<0.001	0.815 (0.808, 0.82)	<0.001
AST	1.027 (1.026, 1.028)	<0.001	1.303 (1.294, 1.313)	<0.001	1.614 (1.59, 1.639)	<0.001	1.614 (1.589, 1.639)	<0.001
ALP	1.006 (1.005, 1.007)	<0.001	1.011 (1.009, 1.012)	<0.001	1.005 (1.003, 1.007)	<0.001	1.005 (1.003, 1.008)	<0.001
γ-GGT	1.001 (1.001, 1.001)	<0.001	0.995 (0.994, 0.996)	<0.001	0.999 (0.998, 1.000)	0.152	0.999 (0.997, 1.000)	0.072
LDL_C	0.918 (0.901, 0.934)	<0.001	1.016 (0.97, 0.941)	0.496	1.043 (0.978, 1.111)	0.199	1.043 (0.977, 1.114)	0.208
HDL_C	2.03 (1.921, 2.145)	<0.001	3.077 (2.675, 3.54)	<0.001	1.246 (1.018, 1.525)	0.033	1.177 (0.953, 1.454)	0.129
Platelet	0.975 (0.975, 0.976)	<0.001	0.952 (0.951, 0.953)	<0.001	0.92 (0.918, 0.922)	<0.001	0.919 (0.918, 0.922)	<0.001
TyG	1.125 (1.099, 1.153)	<0.001	1.636 (1.454, 1.842)	<0.001	0.662 (0.558, 0.787)	<0.001	0.436 (0.224, 0.848)	0.014
TyG_BMI	1.002 (1.001, 1.003)	<0.001	0.996 (0.994, 0.999)	0.018	1.004 (0.999, 1.008)	0.097	1.021 (0.996, 1.048)	0.098
RC	0.948 (0.943, 0.954)	<0.001	0.837 (0.76, 0.922)	<0.001	1.397 (1.218, 1.602)	<0.001	1.336 (1.163, 1.537)	<0.001

SBP, systolic blood pressure; DBP, diastolic blood pressure; BMI, body mass index; ALT, alanine aminotransferase; AST, aspartate aminotransferase; ALP, alkaline phosphatase; γ-GGT, γ-glutamyl transpeptadase; LDL_C, low-density lipoprotein cholesterol; HDL_C, height-density lipoprotein cholesterol; TyG, triglyceride-glucose Index; TyG-BMI, TyG index with body mass index. RC, remnant cholesterol. Model 1, unadjusted; Model 2, adjustment for age and sex; Model 3, adjustment for age; gender; SBP; DBP and BMI.

### 3.3 Univariate and multivariate ordinal logistic analyses of NAFLD by TyG index group

**Figure 3:**
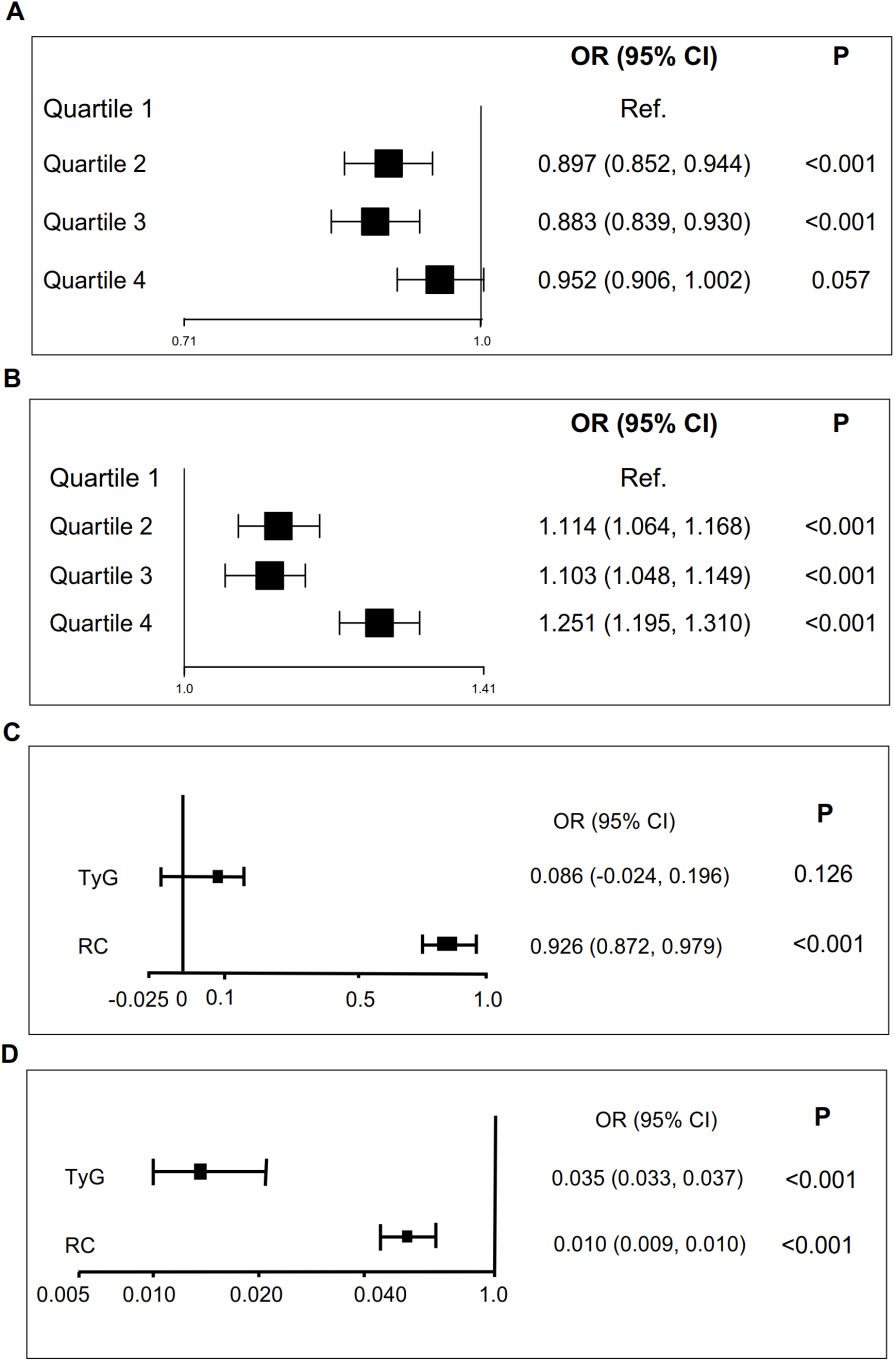
TyG and RC are associated with NAFLD and liver fibrosis scores. **(A)** TyG is associated with NAFLD (adjustment for age, gender, SBP, DBP and BMI). **(B)** RC is associated with NAFLD (adjustment for age, gender, SBP, DBP and BMI). **(C)** TyG is associated with liver fibrosis scores. **(D)**. RC is associated with liver fibrosis scores. .

To further explore the role of TyG index in NAFLD risk stratification, we investigated the effects of different TyG index on the progression of NAFLD (**Table 2**). Patients were divided into four groups based on the quartiles of TyG index (Q1: TyG index ≤ 1.21, Q2: 1.21<TyG index<1.57, Q3: 1.57≤TyG index< 1.97, Q4: TyG index>1.97, **Table 2**). Compared with Q1, Q2 (OR = 0.897 (0.852, 0.944), *P* < 0.001) and Q3 (OR = 0.883 (0.839, 0.930), *P* < 0.001) were associated with a reduction in the progression of NAFLD by 10.3% and 11.7%, respectively (**Table 2**; **Figure 3A**). There was no significant difference between Q4 and Q1 (**Table 2**; **Figure 3A**).

**Table 2:** Association of TyG with the progression of NAFLD.

Characteristics	Mode 1		Model 2		Model 3	
	OR (95% CI)	*P* for trend	OR (95% CI)	*P* for trend	OR (95% CI)	*P* for trend
Quartile 1 (TyG<1.21)	Ref.		Ref.		Ref.	
Quartile 2 (1.21≤TyG<1.57)	1.065 (1.112, 1.221)	0.004	0.899 (0.856, 0.944)	<0.001	0.897 (0.852, 0.944)	<0.001
Quartile 3 (1.57≤TyG<1.97)	1.099 (1.052, 1.147)	<0.001	0.875 (0.834, 0.918)	<0.001	0.883 (0.839, 0.930)	<0.001
Quartile 4 (TyG≥1.97)	1.195 (1.146, 1.247)	<0.001	0.932 (0.890, 0.978)	0.004	0.952 (0.906, 1.002)	0.057

Model 1, unadjusted; Model 2, adjustment for age and sex; Model 3, adjustment for age, gender, SBP, DBP and BMI. Ref.: reference.

### 3.4 Univariate and multivariate ordinal logistic analyses of NAFLD by RC group

Similarly, we also examined how varying RC levels influence the progression of NAFLD (**Table 3**). Participants were divided into four groups according to the quartiles of RC (Q1: RC ≤0.31, Q2: 0.31< RC <0.55, Q3: 0.55≤ RC< 0.88, Q4: RC >0.88, **Table 3**). Compared with Q1, Q2 (OR = 1.114 (1.064, 1.168), *P* < 0.001), Q3 (OR = 1.103 (1.048, 1.149), *P* < 0.001) and Q4 (OR = 1.251 (1.195, 1.310), *P* < 0.001) exacerbated the risk of NAFLD progression by 11.4%, 10.3%, and 25.1%, respectively (**Table 3**; **Figure 3B**).

**Table 3:** Association of RC with the progression of NAFLD.

Characteristics	Model 1		Model 2		Model 3	
	OR (95% CI)	*P* for trend	OR (95% CI)	*P* for trend	OR (95% CI)	*P* for trend
Quartile 1 (RC<0.31)	Ref.		Ref.		Ref.	
Quartile 2 (0.31≤RC<0.55)	1.084 (1.043, 1.127)	<0.001	0.919 (0.880, 0.959)	<0.001	1.114 (1.064, 1.168)	<0.001
Quartile 3 (0.55≤RC<0.88)	1.062 (1.021, 1.103)	0.002	0.893 (0.856, 0.932)	<0.001	1.103 (1.048, 1.149)	<0.001
Quartile 4 (0.31≤RC>0.88)	0.996 (0.958, 1.036)	0.846	1.011 (0.969, 1.055)	0.624	1.251 (1.195, 1.310)	<0.001

Model 1, unadjusted; Model 2, adjustment for age and sex; Model 3, adjustment for age, gender, SBP, DBP and BMI. Ref.: reference.

### 3.5 Sensitivity analyses

ALT, AST, ALP, γ-GGT, LDL-C, HDL-C, platelet, TyG, and RC (excluding TyG_BMI) were found to be associated with the progression of NAFLD (**Table 4**). Multivariate ordinal regression analysis further revealed that AST (OR = 1.149 (1.146, 1.153), *P* < 0.001), HDL-C (1.105 (1.068, 1.142), *P* < 0.001), and RC (OR = 1.114 (1.070, 1.160), *P* < 0.001) exacerbated NAFLD progression (**Table 4**), while ALT (OR = 0.939 (0.937, 0.941), *P* < 0.001), platelet (0.977 (0.977, 0.978), *P* < 0.001), and TyG (OR = 0.904 (0.869, 0.940), *P* < 0.001) can inhibit NAFLD progression (**Table 4**). In addition, we evaluated the impact of different TyG and RC levels on NAFLD progression (**Table 5**). Compared with the Q1 of TyG, the Q2 (OR = 0.635 (0.595, 0.678), *P* < 0.001), Q3 (OR = 0.577 (0.549, 0.607), *P* < 0.001), and Q4 (OR = 0.877 (0.854, 0.901), *P* < 0.001) inhibited NAFLD progression (**Table 5**). Whereas, compared with the Q1 of RC, Q2 (OR = 1.170 (1.089, 1.259), *P* < 0.001), Q3 (OR = 1.116 (1.075, 1.160), *P* < 0.001), and Q4 (OR = 1.063 (1.055, 1.071), *P* < 0.001) exacerbated the risk NAFLD progression (**Table 5**). The consistency of results across both primary and sensitivity analyses reinforces the robustness of our conclusions.

**Table 4:** Determinant factors of NAFLD progression (sensitivity analysis).

Variables	Univariate analysis		Model 1		Model 2		Model 3	
	OR (95% CI)	*P* for trend	OR (95% CI)	*P* for trend	OR (95% CI)	*P* for trend	OR (95% CI)	*P* for trend
Gender	1.423 (1.381, 1.465)	<0001						
Age	1.090 (1.088, 1.091)	<0001						
SBP	1.019 (1.018, 1.020)	<0001						
DBP	1.007 (1.005, 1.008)	<0001						
BMI	0.977 (0.973, 0.982)	<0001						
Albumin	1.002 (1.001, 1.003)	0.003						
ALT	0.995 (0.994, 0.996)	<0001	0.939 (0.937, 0.941)	<0001	0.939 (0.937, 0.941)	<0001	0.939 (0.937, 0.941)	<0001
AST	1.022 (1.021, 1.023)	<0001	1.149 (1.146, 1.153)	<0001	1.149 (1.146, 1.153)	<0001	1.149 (1.146, 1.153)	<0001
ALP	1.007 (1.006, 1.008)	<0001	1.001 (0.999, 1.002)	0.317	1.001 (0.999, 1.002)	0.317	1.001 (0.999, 1.002)	0.287
γ-GGT	1.002 (1.001, 1.002)	<0001	1.000 (1.000, 1.001)	0.657	1.000 (1.000, 1.001)	0.657	1.000 (1.000, 1.001)	0.613
LDL_C	0.920 (0.904, 0.937)	<0001	0.998 (0.975, 1.021)	0.874	0.998 (0.975, 1.021)	0.874	1.001 (0.978, 1.024)	0.942
HDL_C	2.028 (1.919, 2.143)	<0001	1.099 (1.063, 1.135)	<0001	1.099 (1.063, 1.135)	<0001	1.105 (1.068, 1.142)	<0001
Platelet	0.977 (0.977, 0.978)	<0001	0.977 (0.977, 0.978)	<0001	0.977 (0.977, 0.978)	<0001	0.977 (0.977, 0.978)	<0001
TyG	1.050 (1.026, 1.075)	<0001	0.907 (0.872, 0.943)	<0001	0.907 (0.872, 0.943)	<0001	0.904 (0.869, 0.940)	<0001
TyG_BMI	1.000 (0.999, 1.001)	0.598	NA^#^		NA^#^		NA^#^	
RC	0.948 (0.942, 0.955)	<0001	1.106(1.063,1.151)	<0001	1.106(1.063,1.151)	<0001	1.114 (1.070, 1.160)	<0001

SBP, systolic blood pressure; DBP, diastolic blood pressure; BMI, body mass index; ALT, alanine aminotransferase; AST, aspartate aminotransferase; ALP, alkaline phosphatase; γ-GGT, γ-glutamyl transpeptadase; LDL_C, low-density lipoprotein cholesterol; HDL_C, height-density lipoprotein cholesterol; TyG, triglyceride-glucose Index; TyG-BMI, TyG index with body mass index; RC, remnant cholesterol. Model 1, unadjusted; Model 2, adjustment for age and sex; Model 3, adjustment for age, gender, SBP, DBP and BMI. Ref.: reference. #, TyG_BMI was not significant in the univariate analysis, so it was not included in the multivariate analysis.

**Table 5:** Association of TyG and RC with progression of NAFLD (sensitivity analysis).

Characteristics	Model 1		Model 2		Model 3	
	OR (95% CI)	*P* for trend	OR (95% CI)	*P* for trend	OR (95% CI)	*P* for trend
TyG						
Q1	Ref.		Ref.		Ref.	
Q2	1.217 (1.148, 1.289)	<0.001	0.639 (0.599, 0.682)	<0.001	0.635 (0.595, 0.678)	<0.001
Q3	1.131 (1.082, 1.182)	<0.001	0.582 (0.554, 0.611)	<0.001	0.577 (0.549, 0.607)	<0.001
Q4	0.966 (0.944, 0.989)	<0.001	0.869 (0.861, 0.908)	<0.001	0.877 (0.854, 0.901)	<0.001
RC						
Q1	Ref.		Ref.		Ref.	
Q2	1.075 (1.059, 1.092)	<0.001	1.171 (1.089, 1.261)	<0.001	1.170 (1.089, 1.259)	<0.001
Q3	1.070 (1.055, 1.085)	<0.001	1.119 (1.076, 1.164)	<0.001	1.116 (1.075, 1.160)	<0.001
Q4	1.054 (1.047, 1.062)	<0.001	1.063 (1.055, 1.072)	<0.001	1.063 (1.055, 1.071)	<0.001

RC, remnant cholesterol (Q1: RC<0.42, Q2: 0.42 ≤ TyG<0.65, Q3: 0.65 ≤ TyG < 0.82, TyG ≥ 0.82);

TyG, triglyceride-glucose (Q1: TyG<1.26, Q2: 1.21 ≤ TyG<1.57, Q3: 1.52 ≤ TyG < 1.91, TyG ≥ 1.91).

Model 1, unadjusted; Model 2, adjustment for age and sex; Model 3, adjustment for age, gender, SBP, DBP and BMI. Ref., reference.

### 3.6 TyG and RC indices are associated with liver fibrosis scores

To further investigate the role of TyG and RC in liver fibrosis, we examined their associations with non-invasive liver fibrosis scores through linear regression analysis. Both TyG and RC demonstrated positive correlation with liver fibrosis scores, with the exception of the association between the TyG index and the NFS score after adjusting for confounding factors (**Table 6**; **Figure 3**). The increase in TyG by 1-unit were associated with a corresponding elevation of 0.035 in the APRI score (β = 0.035 (0.033, 0.037)), and the increase of RC by 1-unit, NFS score (β = 0.926 (0.872, 0.979), **Table 6**; **Figure 3C**) and APRI score (β = 0.010 (0.009, 0.01), **Table 6**; **Figure 3D**) increased by 0.979 and 0.0110, respectively.

**Table 6:** TyG and RC are associated with non-invasive liver fibrosis score.

Variables	Model 1		Model 2		Model 3	
	β (95% CI)	*P*	β (95% CI)	*P*	β (95% CI)	*P*
NFC score						
TyG	0.407 (0.271, 0.544)	<0.001	0.207 (0.088, 0.326)	0.001	0.086 (-0.024, 0.196)	0.126
RC	0.791 (0.725, 0.857)	<0.001	0.761 (0.703, 0.819)	<0.001	0.926 (0.872, 0.979)	<0.001
APRI score						
TyG	0.035 (0.034, 0.037)	<0.001	0.039 (0.037, 0.041)	<0.001	0.035 (0.033, 0.037)	<0.001
RC	0.011 (0.010, 0.012)	<0.001	0.010 (0.010, 0.011)	<0.001	0.010 (0.009, 0.010)	<0.001

NFS, non-invasive fibrosis score; APRI, aspartate to platelet ratio index; TyG, triglyceride-glucose Index; RC remnant cholesterol.

Model 1, unadjusted; Model 2, adjustment for age and sex; Model 3, adjustment for age, gender, SBP, DBP and BMI.

## 4 Discussion

The TyG index and remnant cholesterol (RC), indicative of insulin resistance and dyslipidemia, are established risk factors for NAFLD (1, 3, 11). Our study identified significant associations between NAFLD and serum levels of ALT, AST, ALP, TyG, and RC. Interestingly, elevated TyG levels were unexpectedly correlated with an inhibition of NAFLD progression, whereas higher RC levels were linked to increase progression. When patients were stratified into quartiles based on TyG and RC levels, these associations persisted across all subgroups. Both TyG and RC also showed positive correlations with validated non-invasive markers of liver fibrosis (NFS and APRI scores).

Traditional dyslipidemia, characterized by elevated triglycerides, increased LDL-C, and reduced HDL-C, has long been implicated in the pathogenesis of NAFLD (17, 18). RC, representing the cholesterol component of triglyceride-rich lipoproteins, independently predicts adverse cardiovascular outcomes beyond conventional lipid parameters (9, 19). Prospective cohort evidence suggests that RC reducing RC levels more effectively decreases major cardiovascular events compared to similar reductions in LDL-C or non-HDL-C (20), while cross-sectional studies have associated elevated RC with an increased risk of NAFLD in both adults and adolescents (14, 21). These findings position RC as a potentially more informative biomarker than traditional lipid measures for NAFLD. Our large-sample cross-sectional study provided novel evidence that serum RC levels are correlated with all stages of NAFLD progression (mild, moderate, and severe), with these associations remaining significant after adjusting for age, gender, SBP, DBP, and BMI. In addition, multicollinearity diagnostics were performed for independent variables prior to conduct multivariate ordinal regression, and the results indicated no significant multicollinearity concerns, thereby confirming the stability and reliability of our findings. Recent cohort data corroborate this, showing the highest RC quartile have a greater risk of developing NAFLD compared to those in the lowest quartile, even among patients with normal levels of LDL-C, HDL-C, and triglycerides (19). Our analysis further quantified a 10–25% increased risk of NAFLD in the second through fourth RC quartiles compared to the first, a finding facilitated by our large-scale cross-sectional design.

Additionally, this study presents a linear regression analysis evaluating the association between serum RC levels and liver fibrosis, revealing that elevated serum RC is a positively associated with NFS/APRI scores, indicating its potential role in fibrotic progression during liver disease. While the mechanisms connecting RC to the development of NAFLD remain not fully elucidated, three potential pathways may mediate this association. Activation of hepatic stellate cell is facilitated by RC-induced TLR4/NF-κB signaling, which in turn stimulates the secretion of TGF-β1 and subsequent collagen deposition (22). RC also promotes the activation of NLRP3 inflammasome, thereby exacerbating insulin resistance-related inflammation through the upregulation of IL-1β (22). Additionally, RC-modified LDL_C leads to a reduction in sulfatide content, impairing the function of NKT cells and compromising immune surveillance within the hepatic microenvironment (23). Future studies should utilize longitudinal cohorts with histological confirmation to quantify the dynamics between RC and insulin resistance across the spectrum of NAFLD, from steatosis to advanced fibrosis. The development of predictive algorithms that incorporate RC-insulin resistance indices could improve the accuracy of risk stratification.

Beyond RC, the TyG index, an emerging biomarker of IR, has also been associated with NAFLD in previous studies (1, 12). However, these investigations were limited by their focus on general populations, limited sample sizes, and lack of longitudinal RC data across different stages of NAFLD, which constrained their conclusions (12, 13). n our comprehensive analysis employing of a larger cross-sectional design, we unexpectedly identified the TyG index as a protective factor against the progression of NAFLD following multivariate adjustment, a finding that contradicts g existing evidence. This phenomenon is intriguing and not uncommon in scientific research. The key findings of this study remained consistent after adjusting for potential confounding factors, conducting sensitivity analyses, and evaluating multicollinearity. The observed discrepancies could be interpreted from several perspectives. First, previous studies have associated the TyG index with the incidence of NAFLD, and NAFLD is often accompanied by other chronic diseases (4, 8, 12, 13), whereas our investigation focused on the progression of NAFLD in patients with NAFLD alone, which may involve a distinct pathophysiological process. Second, previous studies involved severely ill populations (4, 8, 12, 13), whereas our cohort consisted of relatively healthier individuals. In high-risk groups, elevated TyG levels may be prevalent, whereas in our healthier participants, what is considered “high” may still fall within a metabolically adaptive range. Third, the disparity in data distribution across subgroups (75,414 mild cases, 19,412 moderate cases, and 7,733 severe cases) may have contributed to the discrepancies between our findings and those of previous studies. In addition, the effect of TyG may vary with age and gender. In younger individuals, a robust insulin response could imply that an elevated TyG index reflects metabolic activity rather than dysfunction. Although we adjusted for age and sex, (acknowledging that mild cases tended to be younger and included fewer females) this inherent imbalance among groups may have influenced the results. The established association between the TyG and NAFLD may involve three mechanisms. First, hepatic triglyceride accumulation exacerbates insulin resistance, leading to elevated fasting glucose and triglyceride-rich LDL levels (24). Second, obesity and metabolic syndrome (24), where excessive energy intake increases hepatic fat deposition and free fatty acids, which are key precursors of triglyceride (25). Third, the TyG is also associated with muscle insulin resistance (26), potentially redirecting glucose flux to the liver and promoting lipid accumulation. Therefore, the relationship between the TyG index and disease outcomes may not be universal but rather context-dependent. Our study underscores the importance of considering patient-specific factors in the interpretation of this biomarker. Future prospective studies in similar populations, along with mechanistic investigations, are necessary to validate this paradoxical association and to elucidate its underlying mechanisms. ‘While elevated TyG index quartiles are predictive of NAFLD prevalence and incidence, their association with the progression of liver fibrosis in NAFLD patients remains inadequately understood. Previous studies linking TyG-related parameters to fibrosis exhibited critical methodological limitations, such as the incorrect reversal of independent (TyG) and dependent (fibrosis score) variables (27, 28), thus hindering causal inference. In this study, we report the clinical evidence of a significant positive correlation between elevated TyG index and liver fibrosis severity, as measured by validated biomarkers (NFS and APRI). Our findings found that, although the medium-to-high TyG levels showing a protective effect against NAFLD progression, the TyG index was positively associated with liver fibrosis scores. These scores, however, are limited by potential for indeterminate results and a low positive predictive value, indicating that elevated scores cannot definitively confirm the presence of advanced fibrosis. Furthermore, the NFS score may be confounded by extrahepatic conditions that affect AST or platelet levels and is inherently incapable of providing information on other critical histological features of nonalcoholic steatohepatitis (NASH), such as inflammation. Therefore, future studies should investigate the utility of the TyG index in monitoring fibrosis regression therapy, assess its predictive value in patients with comorbid metabolic disorders, and explore its integration with inflammatory markers (e.g., platelet count) and anthropometric parameters (e.g., waist circumference) to enhance risk stratification and metabolic interventions.

This study has several limitations that warrant consideration. First, the retrospective design precludes causal interpretation of the TyG-NAFLD relationship, necessitating validation through prospective studies with serial measurements. Second, although NFS and APRI have important roles in large-scale population screening and in identifying individuals at low risk, it is recommended that future research incorporate comprehensive histological assessments or more precise imaging-based elastography techniques utilized in specialized clinical settings. Third, the key findings remained significant after conducting sensitivity analyses, adjusting for covariate, and assessing multicollinearity. However, the protective effect of TyG index against the progression of NAFLD requires cautious interpretation, and future studies with improved prospective designs are necessary to further elucidate the role of the TyG index in the progression of NAFLD. Finally, the generalizability of our findings is limited by the exclusively Chinese cross-sectional study design; therefore, validation in in multiethnic populations, particularly those with distinct metabolic risk profiles (e.g., European and North American), is essential to confirm these results.

## 5 Conclusion

Serum RC and the TyG index demonstrated significant correlations with NAFLD severity across mild, moderate, and severe stages, showing consistent positive associations with NFS and APRI scores. Our findings suggest that the TyG index and RC may serve as independent predictors of NAFLD progression and hepatic fibrosis, highlighting their potential utility in risk stratification and the development of targeted therapeutic strategies.

## Data Availability

Data are available from the corresponding author (Xu Zhang: zhangxu2673@163.com) upon reasonable request.
