# Supplementary material for: Association of triglyceride-glucose index and remnant cholesterol levels with liver fibrosis progression and disease severity in patients with non-alcoholic fatty liver disease: a cross-sectional study
Source: Front Endocrinol (Lausanne). 2025 Nov 24;16:1686162. doi: 10.3389/fendo.2025.1686162 (PMC12684284; doi:10.3389/fendo.2025.1686162)
Supplement: Supplementary file 1 [file Table1.docx]

**Supplementary Table1 Demographic and clinical characteristics of NAFLD patients** **(n=102559)**

|  | Mild  (n=75414) | Moderate  (n=19412) | Severe  (n=7733) | Moderate *vs*. Mild | |  | Severe *vs*. Mild | |  | Severe *vs*. Moderate | |
| --- | --- | --- | --- | --- | --- | --- | --- | --- | --- | --- | --- |
|  |  |  |  | Z/χ^2^ | *P* |  | Z/χ^2^ | *P* |  | Z/χ^2^ | *P* |
| Gender (Female, %) | 19355 (25.7) | 6869 (35.4) | 2265 (29.3) | 729.1 | <0.001 |  | 47.9 | <0.001 |  | -92.0 | <0.001 |
| Age (years) | 43.0 (35.0, 53.0) | 60.0 (54.0, 68.0) | 53.0 (41.0 ,62.0) | -154.1 | <0.001 |  | -51.4 | <0.001 |  | -43.6 | <0.001 |
| Height (cm) | 171.0 (165.0, 176.0) | 167.0 (160.0, 172.0) | 169.0 (162.0, 174.0) | -56.1 | <0.001 |  | -19.9 | <0.001 |  | -14.8 | <0.001 |
| Weight (kg) | 75.0 (68.0, 83.0) | 71.0 (64.0, 78.4) | 73.0 (66.0, 81.0) | -43.6 | <0.001 |  | -12.9 | <0.001 |  | -13.7 | <0.001 |
| BMI (kg/m^2) | 25.8 (24.0, 27.9) | 25.6 (23.8, 27.6) | 25.9 (24.0, 27.9) | -9.9 | <0.001 |  | -0.7 | 0.499 |  | -6.4 | <0.001 |
| SBP (mmHg) | 128.0 (118.0, 139.0) | 136.0 (124.0, 149.0) | 132.0 (120.0, 145.0) | -49.9 | <0.001 |  | -15.4 | <0.001 |  | -14.9 | <0.001 |
| DBP (mmHg) | 80.0 (72.0, 88.0) | 82.0 (74.0, 90.0) | 80.0 (72.0, 88.0) | -17.1 | <0.001 |  | -0.6 | 0.542 |  | -10.4 | <0.001 |
| ALT (U/L) | 28.0 (20.0, 42.0) | 24.0 (18.0, 35.0) | 33.0 (20.0, 70.0) | -32.3 | <0.001 |  | -12.5 | <0.001 |  | -21.3 | <0.001 |
| AST (U/L) | 22.0 (18.0, 27.0) | 24.0 (20.0, 30.0) | 27.0 (20.0, 44.0) | -49.9 | <0.001 |  | -38.1 | <0.001 |  | -16.8 | <0.001 |
| ALP (U/L) | 77.0 (65.0, 92.0) | 80.0 (68.0, 96.0) | 79.0 (66.0, 94.0) | -13.2 | <0.001 |  | -4.1 | <0.001 |  | -4.2 | <0.001 |
| γ-GGT (U/L) | 33.0 (22.0, 54.0) | 30.0 (20.0, 51.0) | 33.0 (21.0, 54.0) | -9.7 | <0.001 |  | -0.3 | -0.800 |  | -5.4 | <0.001 |
| TG (mmol/l) | 1,7 (1.2, 2.5) | 1.7 (1.2, 2.4) | 1.7 (1.2, 2.5) | -4.5 | <0.001 |  | -0.1 | 0.901 |  | -2.7 | 0.008 |
| Glucose (mmol/l) | 5.4 (5.1, 5.9) | 5.7 (5.3, 6.4) | 5.5 (5.1, 6.1) | -49.1 | <0.001 |  | -4.7 | <0.001 |  | -20.8 | <0.001 |
| TC (mmol/l) | 4.9 (4.3, 5.5) | 4.9 (4.3, 5.6) | 5.0 (4.4, 5.7) | -3.5 | <0.001 |  | -7.6 | <0.001 |  | -4.9 | <0.001 |
| LDL_C (mmol/l) | 3.1 (2.5, 3.6) | 3.0 (2.5, 3.6) | 3.0 (2.5, 3.6) | -8.6 | <0.001 |  | -6.0 | <0.001 |  | -0.3 | 0.772 |
| HDL_C (mmol/l) | 1.2 (1.0, 1.3) | 1.2 (1.1, 1.4) | 1.2 (1.0, 1.4) | -30.8 | <0.001 |  | -9.9 | <0.001 |  | -7.3 | <0.001 |
| Platelet (×10^9/l) | 246.0 (214.0, 283.0) | 183.0 (158.0, 211.0) | 195.5 (132, 248.0) | -148.4 | <0.001 |  | -60.6 | <0.001 |  | -7.6 | <0.001 |
| Albumin (g/l) | 46.3 (44.2, 48.2) | 45.4 (43.4, 47.2) | 45.8 (43.9, 47.4) | -34.5 | <0.001 |  | -11.9 | <0.001 |  | -7.8 | <0.001 |
| NFS | -30.0 (-31.8, -27.5) | -27.7 (-29.2, -25.9) | -25.6 (-27.4, -3.8) | -85.0 | <0.001 |  | -51.4 | <0.001 |  | -32.4 | <0.001 |
| APRI | 0.3 (0.2, 0.3) | 0.4 (0.3, 0.5) | 0.3 (0.0, 0.6) | -127.8 | <0.001 |  | -4.1 | <0.001 |  | -31.1 | <0.001 |
| TyG | 1.6 (1.2, 2.0) | 1.6 (1.2, 2.0) | 1.6 (1.2, 2.0) | -9.1 | <0.001 |  | -4.3 | <0.001 |  | -1.1 | 0.259 |
| TyG_BMI | 38.4 (26.5, 50.6) | 38.8 (27.0, 51.1) | 39.9 (28.4, 52.1) | -2.7 | 0.006 |  | -6.4 | <0.001 |  | -4.2 | <0.001 |
| RC (mmol/L) | 0.6 (0.3, 0.9) | 0.6 (0.3, 0.9) | 0.5 (0.2, 0.9) | -3.2 | 0.001 |  | -8.7 | <0.001 |  | -9.3 | <0.001 |

BMI body mass index, SBP systolic blood pressure, DBP diastoic blood pressure, ALT alanine aminotransferase, AST asprartate aminotransferase, ALP alkaline phosphatase, γ-GGT γ-glutamyl transpeptadase, RC remnant cholesterol, TG triglycerides, TC total cholesterol, TyG triglyceride-glucose, LDL_C low-density lipoprotein cholesterol, HDL_C hight-density lipoprotein cholesterol, NFS non-invasive fibrosis score, APRI aspartate to platelet ratio index.

**Supplementary Table 2. Multicollinearity diagnostics (VIF and Tolerance)**

|  | Collinearity statistics | |
| --- | --- | --- |
|  | Tolerance | VIF |
| ALT | 0.390 | 2.561 |
| AST | 0.405 | 2.472 |
| ALP | 0.936 | 1.069 |
| γ-GGT | 0.773 | 1.293 |
| HDL_C | 0.774 | 1.292 |
| LDL_C | 0.827 | 1.210 |
| RC | 0.371 | 2.694 |
| Platelet | 0.971 | 1.030 |
| TyG | 0.225 | 4.435 |
| TyG_BMI | 0.371 | 2.692 |

ALT alanine aminotransferase, AST asprartate aminotransferase, ALP alkaline phosphatase, APRI aspartate to platelet ratio index, γ-GGT γ-glutamyl transpeptadase, LDL_C low-density lipoprotein cholesterol, HDL_C hight-density lipoprotein cholesterol, NFS non-invasive fibrosis score, RC remnant cholesterol, TG triglycerides, TC total cholesterol, TyG triglyceride-glucose.

**Supplementary Table 3. Multicollinearity diagnostics (Eigenvalue and Condition index)**

| Dimension | Eigenvalue | Condition index | ALT | AST | ALP | GGT | HDL_C | LDL_C | RC | Platelet | TyG | TyG_BMI |
| --- | --- | --- | --- | --- | --- | --- | --- | --- | --- | --- | --- | --- |
| 1 | 9.081 | 1 | 0 | 0 | 0 | 0 | 0 | 0 | 0 | 0 | 0 | 0 |
| 2 | 0.655 | 3.724 | 0.07 | 0.06 | 0 | 0.15 | 0 | 0 | 0.01 | 0 | 0 | 0 |
| 3 | 0.512 | 4.21 | 0.01 | 0.01 | 0 | 0.13 | 0.01 | 0.01 | 0.12 | 0.01 | 0 | 0.01 |
| 4 | 0.349 | 5.098 | 0.06 | 0.05 | 0 | 0.67 | 0 | 0 | 0.05 | 0 | 0 | 0 |
| 5 | 0.109 | 9.116 | 0.18 | 0.21 | 0.01 | 0 | 0.01 | 0.01 | 0.3 | 0 | 0.01 | 0.27 |
| 6 | 0.091 | 9.967 | 0.58 | 0.57 | 0 | 0 | 0 | 0 | 0.11 | 0.06 | 0 | 0.14 |
| 7 | 0.069 | 11.507 | 0.01 | 0.02 | 0.77 | 0.01 | 0.01 | 0.07 | 0.01 | 0.1 | 0 | 0 |
| 8 | 0.057 | 12.622 | 0.05 | 0.04 | 0.01 | 0 | 0.08 | 0.24 | 0.05 | 0.6 | 0 | 0.02 |
| 9 | 0.042 | 14.786 | 0.04 | 0.03 | 0.05 | 0 | 0.38 | 0.59 | 0.01 | 0.02 | 0 | 0.07 |
| 10 | 0.024 | 19.534 | 0 | 0 | 0.05 | 0 | 0.01 | 0.08 | 0.34 | 0.01 | 0.82 | 0.47 |
| 11 | 0.011 | 29.205 | 0 | 0 | 0.1 | 0.03 | 0.49 | 0 | 0.01 | 0.2 | 0.16 | 0.01 |

ALT alanine aminotransferase, AST asprartate aminotransferase, ALP alkaline phosphatase, APRI aspartate to platelet ratio index, γ-GGT γ-glutamyl transpeptadase, LDL_C low-density lipoprotein cholesterol, HDL_C hight-density lipoprotein cholesterol, NFS non-invasive fibrosis score, RC remnant cholesterol, TG triglycerides, TC total cholesterol, TyG triglyceride-glucose.
